# Clinical Lectures on Diseases of the Eye

**Published:** 1863-11

**Authors:** E. L. Holmes

**Affiliations:** Chicago, Lecturer on Diseases of the Eye and Ear in Rush Medical College and Surgeon of the Chicago Charitable Eye and Ear Infirmary


### CLINICAL LECTURES ON DISEASES OF THE EYE.

By E. L. HOLMES, M. D., of Chicago,

*Lecturer on Diseases of the Eye and Ear in Rush Medical College and Surgeon of the Chicago Charitable Eye and Ear Infirmary.*

### INTRODUCTION.

GENTLEMEN:

In entering upon a course of clinical study of the diseases of the eye and ear and their treatment, it may be well for you to spend a few moments in considering what advantages you are to derive from the time and labor such a course demands.

To a class of young men, who are pledged, as it were, to devote their whole energies and strength to the search for the means and the opportunities of relieving and preventing human suffering, it might seem more than useless to waste time in considering these advantages, almost self evident; for who among you cannot fully weigh the inestimable value of the delicate organ we propose to study; who can fail to appreciate the terrible condition of the unfortunate blind, doomed to grope their way through a life of darkness; who would not feel the deepest interest in seeking the means of restoring such sufferers to the light of day?

And yet, gentlemen, the restoration of the blind to sight, noble and crowned with brilliant success as the efforts for such a result sometimes are, is not practically a subject of the greatest importance for your consideration at this time. It is without doubt of more importance for you to seek the means of preventing blindness by the judicious treatment of very many diseases which too often, if neglected or improperly treated, result in total or partial loss of sight; or, at least, although they scarcely attract the notice of the casual observer, bring with them much pain and unhappiness, and render their victims unable to perform the common duties of life. Such patients are among the most pitiful objects that come to us for aid and advice.

One would naturally suppose, in view of all this, that so important an organ as the eye, which in every community, especially in the Western States, is subject to a great variety of diseases, would excite the interest of every student about to commence the study of medicine, and of every practitioner in active business, meeting, as he does, numerous and sad examples of these diseases.

But this is not the case. Little has hitherto been done to encourage the student to secure a knowledge of the diagnosis and treatment of Ophthalmic disease; even practitioners usually neglect the study and too often confess not only their ignorance of the subject, but also their dislike to treat patients afflicted with diseases of the eye. Do not misunderstand me. I do not wish to come before you to criticise flippantly the course of study, which has been followed by nearly all the eminent members of our profession of the past and present, and which, all circumstances considered, has been deemed by our most distinguished teachers of medicine in our country the most practical which the student could pursue. I would scarcely venture to dwell upon the neglect of Ophthalmic science on the part of so large a proportion of our profession were it not that the neglect is almost universally admitted.

Few can estimate the extent of evil arising in every community from the ignorance of medical attendants in the treatment of affections of the eye. No small number of patients in this State alone are yearly doomed, from this cause, to long agonizing sickness, terminating in blindness or irreparable diminution of sight, which renders them more or less dependent for life upon others for support, and which increases pauperism and ignorance and even vice among our populace. Fortunately, the consciousness of this ignorance and the painful prevalence of the evils, of which I have just spoken, as its result, have awakened an almost universal desire that better opportunities should be provided for the student to pursue a systematic course of clinical instruction in diseases of the eye. Already, in several cities of our country, greater facilities for this study are afforded the student, and in a few cases lecturers on Ophthalmology have been appointed in our medical schools. In many of the most celebrated medical colleges of Europe, Ophthalmology has long been the subject of special instruction. Teachers eminently qualified for their positions are appointed, and the student is not only encouraged to pursue the study, but is obliged to pass a special examination in this department of medical science, previous to taking his degree.

Now, gentlemen, I only wish to present to you the claims which Ophthalmology has upon you for your most earnest attention—not to set it above other branches of our science.

You are expected to prepare yourself creditably for the active duties of your profession, whether it be in the management of general surgical and medical diseases, or of the lying-in room. Whether you prepare yourself or not for the treatment of diseases of the eye, the public, except in large cities, will turn to you for advice in cases of disease of this most beautiful and most important organ. You will probably, in general country practice, be called far oftener to treat diseased eyes than to reduce a dislocation, to adjust a fractured limb, or to treat a case of typhoid fever or pneumonia. It must not be imagined that practitioners, who are eminently qualified to perform their duties as surgeons, as physicians, or as obstetricians, are able, as is sometimes said, from a knowledge of the general principles of disease, to understand and treat diseases of the eye. Ask any of your friends who have had an experience, however long, in the general practice of medicine, if they are willing to trust their judgment in the diagnosis and treatment of diseases of the eye; they will invariably tell you no, unless they have had especial opportunities of observing the course of such diseases. While diseases of the eye have their analogues in other organs of the system, it requires long study and observation to acquire skill in recognizing the progress of disease in the different tissues of so delicate an organ.

Therefore, I repeat, those of you who intend to practice in the country, owe it as a great duty to yourselves and your patients to be prepared to treat ordinary diseases of the eye. And, I repeat, you will meet with inflammatory diseases of the eye in many parts of our country more frequently than that of any other organ. I have been informed by several intelligent practitioners, that ophthalmic diseases form, at certain seasons of the year, nearly a quarter of all the cases to which they are called. And I would ask, in reference to the relative importance of this subject, who would not prefer to carry through life a crooked limb as the result of ignorance in the treatment of a fracture, than from a similar cause, to pass his days with injured if not extinguished vision. I have seen sad results from neglect and ignorance on the part of the attending surgeon in the treatment of quite a large number of fractures. But sad, as some of these cases have been, I must say no one of them can equal the terrible calamity, which I have often seen, borne for life by the poor victims of improper treatment of diseased eyes. I do not wish you to study any department of general surgery or medicine the less, but to impress upon your minds the duty you have of preparing yourselves for treating ophthalmic disease more than students usually have done.

I wish to ask your attention to what I conceive to be just views, regarding specialists and specialties, as considerable prejudice on this subject exists in the minds of many men, eminent in our profession, especially in this country. In Europe the prejudice exists to some extent, but is fast yielding to an enlightened appreciation of the demands of modern science. The science of medicine is already too broad for the general practitioner, occupied by his active duties, to be thoroughly educated in every branch. What member of our profession, however studious he may have been, is there, whose judgment would be reliable alike in difficult cases of surgical, medical, obstetric and ophthalmic practice? To whom in our large cities or in the country do even our best educated general practitioners turn for advice, in cases of diseases of the skin, of the chest, of the eye and the ear, of insanity and syphilitic disease? Most certainly to those who have thought most on these respective subjects, who have studied them most and have had the best opportunities to observe the largest number of cases. Even those who are most prejudiced against specialists, in spite of their prejudice most naturally turn to the same class of men for counsel. They cannot deny that the standard authority in each of the departments of medicine and surgery are the works of men, who are specialists in their respective department.

What medical works do the teachers of our medical colleges place in the hands of their students as text-books, or as authorities for subsequent study and reference, but those, prepared by men, who have concentrated their principal efforts of mind upon one class of subjects?

Gentlemen, our science, like every science, is simply a knowledge of facts, and all progress in science consists in the discovery of facts; and the discovery of facts, important facts, is one of the most difficult tasks required of the human mind. As far as I know, in every branch of our studies, ophthalmology as well as general surgery and medicine, facts have been established by the labor and observation of those who have made a special study of the diseases of particular organs.

The same individuals who seem most opposed to specialists, are practically, to a certain extent, compelled to acknowledge the advantage of making a special study one subject of disease or one class of diseases.

And I apprehend, few men in our profession are really opposed to the scientific pursuit of specialties. The prejudice has undoubtedly its origin and support in the fact that the character of so-called specialists, has almost universally been unworthy the noble profession they have entered; they have been unprincipled and ignorant charlatans, who make the public journals a medium by which they spread before the world their pretentious advertisements, full of false promises and impudent arrogance, which are listened to by the afflicted, who, as a result of their misplaced confidence, are not only ruined in health, but robbed of their limited means of support.

Such pretenders are usually as ignorant of the diseases they profess invariably to cure, as they are unscrupulous in their means of extorting money. Whenever, gentlemen, you read in the public journals, the extravagant advertisements of those who profess to possess extraordinary skill in the treatment of the diseases of any special organ, be sure the author of the advertisement is dishonest: his sole aim is to prey upon the means of the suffering and afflicted, to secure which he will trifle with the lives and health of his unfortunate dupes.

To a certain extent, the profession is responsible for the existence of so much charlatanry, especially in treating diseases of the eyes. Were physicians generally less ignorant in the management of even ordinary diseases of the eye, the public would place more confidence in their judgment, and would certainly listen less eagerly to one class of impostors, traveling oculists.

No one, however, even those most scrupulous on this point, can object to the course adopted by some of the most distinguished writers in our profession, who, after preparing themselves carefully in every department, have chosen one to which they have subsequently devoted their time and labors. It is undoubtedly true, that no one can be so well fitted for the most satisfactory study of special diseases as one who has well studied disease in general, as the student, whose preparatory studies have embraced a wise course of language, literature and science, is best qualified to commence the study of general medicine and surgery. So, gentlemen, if any of you intend to make diseases of the eye one of the principal studies of your life, let me earnestly advise you to pursue with diligence every branch to which your Professors invite your attention.

We are about to enter upon the study of one of the most interesting and yet neglected subjects in the science of medicine. It embraces the anatomy, physiology and pathology of the eye, with all that is known of the beautiful laws of light. The field of study is a wide one—wider than the most of you probably imagine.

The present course, from circumstances which you all understand, must necessarily be somewhat limited in scope. But I shall feel that I have accomplished much, if I can so interest you in the subject, that acquiring such grains of knowledge as you may be able during the coming term of study, you will hereafter continue your labors and perfect yourselves in all that pertains to the treatment of the diseases of the eye.

A few words in regard to text-books. I am sorry to say that in English there is not a single tex-book containing all that is known at the present time of the anatomy, physiology and pathology of the different tissues of the eye. From time to time, as we study the different diseases, I will call your attention to the best authorities. But let me allude especially to the writings of Graefe, of Berlin, and Donders, of Utrecht, in Holland.

The best general works for study and reference are, perhaps, in German, those of Stellwag and Arlt; in English, those of Lawrence and Mackenzie. Possibly, in view of your present limited time for study, the smaller handbooks of Dixon and Williams, although very superficial in some highly important points, may serve your purpose for the present better than any works you can readily obtain.

I would especially call your attention to the *American Journal of Ophlhalmology*, edited by Dr. Homberger, of New York. This journal will inform you from time to time of all important advances in the treatment of diseases of the eye. It will give you reviews of new works and translations of valuable monographs. The first volume, just completed, contains a translation of Graefe's monograph on Acute Conjunctivitis and the use of Caustics, which alone, to those of you who cannot obtain the original article, is worth more than the cost of the volume.

